# IFNα and 5-Aza-2’-deoxycytidine combined with a dendritic-cell targeting DNA vaccine alter tumor immune cell infiltration in the B16F10 melanoma model

**DOI:** 10.3389/fimmu.2022.1074644

**Published:** 2023-01-19

**Authors:** James T. Gordy, Avinaash K. Sandhu, Kaitlyn Fessler, Kun Luo, Aakanksha R. Kapoor, Samuel K. Ayeh, Yinan Hui, Courtney Schill, Fengyixin Chen, Tianyin Wang, Styliani Karanika, Joel C. Sunshine, Petros C. Karakousis, Richard B. Markham

**Affiliations:** ^1^ Department of Molecular Microbiology and Immunology, Johns Hopkins Bloomberg School of Public Health, Baltimore, MD, United States; ^2^ Division of Infectious Diseases, Center for Tuberculosis Research, Department of Medicine, The Johns Hopkins Hospital, Baltimore, MD, United States; ^3^ The Departments of Dermatology, Pathology, and Biomedical Engineering, Johns Hopkins University School of Medicine, Baltimore, MD, United States

**Keywords:** interferon, 5-Aza-2’-deoxycitidine, dendritic cell, vaccine, B16F10 melanoma, chemokine, CCL20, CD8+ T-cells

## Abstract

**Introduction:**

DNA vaccines containing a fusion of the gene encoding chemokine MIP-3α (CCL20), the ligand for CCR6 on immature dendritic cells (DCs), to melanoma-associated antigen genes have enhanced anti-tumor immunity and efficacy compared to those lacking the chemokine gene. Previous work has shown that type-I interferon (IFNα or IFN) and 5-Aza-2’-deoxycytidine (5Aza) significantly enhance the therapeutic benefit of DNA vaccines as measured by reduced tumor burden and improved mouse survival.

**Methods:**

Here, we explored mouse intratumoral immune correlates underlying the therapeutic benefit of this combination regimen (vaccine, IFN, and 5Aza) as compared to vaccine alone and IFN and 5Aza without vaccine, focusing on chemokine mRNA expression by qRT-PCR and inflammatory cellular infiltration into the tumor microenvironment (TME) by flow cytometry and immunohistochemistry (IHC).

**Results:**

The combination group significantly upregulated intratumoral mRNA expression of key immune infiltration chemokines XCL1 and CXCL10. Flow cytometric analyses of tumor suspensions exhibited greater tumor infiltration of CD8+ DCs, CCR7+ DCs, and NK cells in the combination group, as well as reduced levels of myeloid-derived suppressor cells (MDSCs) in vaccinated groups. The mice receiving combination therapy also had greater proportions of effector/memory T-cells (Tem), in addition to showing an enhanced infiltration of Tem and central memory CD8+ T-cells, (Tcm). Tem and Tcm populations both correlated with smaller tumor size. Immunohistochemical analysis of tumors confirmed that CD8+ cells were more abundant overall and especially in the tumor parenchyma with combination therapy.

**Discussion:**

Efficient targeting of antigen to immature DCs with a chemokine-fusion vaccine offers a potential alternative approach to classic and dendritic cell-based vaccines. Combining this approach with IFNα and 5Aza treatments significantly improved vaccine efficacy. This treatment creates an environment of increased inflammatory chemokines that facilitates the trafficking of CD8+ DCs, NK cells, and CD8+ T-cells, especially memory cells, while reducing the number of MDSCs. Importantly, in the combination group, CD8+ cells were more able to penetrate the tumor mass in addition to being more numerous. Further analysis of the pathways engaged by our combination therapy is expected to provide additional insights into melanoma pathogenesis and facilitate the development of novel treatment strategies.

## Introduction

Despite advances in medical innovation and treatment, cancer resulted in nearly 10 million deaths globally in 2020 (1). Of these deaths, over 57,000 were from melanoma (2). Traditional treatments like surgery and chemotherapy aid in early stages of the disease but treating late-stage metastatic melanoma remains a challenge. Recently, immunotherapies such as immune checkpoint blockades (ICB) targeting the markers CTLA-4 and PD-1/PD-L1 have shown promise, but their use is limited by the severity of their associated side effects and a high frequency of non-responsiveness and relapse (3). The limited success of these treatments has prompted the deployment of combination therapies, which often include traditional anticancer drugs such as decitabine and immunotherapeutic agents such as interferon-α, ICB, and CAR-T-cells (3–6).

Two bottlenecks in the development of cancer immunotherapy are activation of sufficient numbers of effector cells targeting tumor antigens and ensuring that those effector cells enter the tumor environment. Cancer vaccines have been employed to expand populations of cancer antigen specific T-cells (7) frequently employing approaches that recognize the importance of dendritic cell (DC) recruitment in T-cell activation (8, 9). We have previously reported marked enhancement of anti-tumor efficacy of a DC-targeting melanoma vaccine by the addition of Interferon α (IFN) and 5’aza-2 deoxycytidine (5Aza) to the therapeutic regimen. Of note, the enhanced efficacy of this regimen was dependent on the presence of all components and was not attributable to additive effects of individual components. In the current studies we have explored the intratumoral immune parameters of the regimen components IFN+5Aza, Vaccine, and the combination to define their roles in overcoming the treatment bottlenecks. Our findings provide a basis for understanding the requirement for all treatment components to achieve the synergistic efficacy observed with this treatment regimen.

This study investigated the intratumoral immune mechanisms associated with the enhanced therapeutic efficacy of combinatorial treatment seen in previous studies (10, 11). In line with previous findings, CD8+ T-cells were enriched in tumors from mice receiving combination therapy relative to mice receiving IFN and 5Aza or mice receiving vaccine alone. Chemokines important for attracting inflammatory cells, such as CCL19 (11), CXCL10, and XCL1, were significantly upregulated. Inflammatory cell types such as natural killer (NK) cells, CD8+ DCs, and memory CD8+ T-cells were also significantly enriched, whereas levels of myeloid-derived suppressor cells (MDSCs) were greatly reduced. Importantly, the IHC results highlight that the CD8+ cells in the combination group are of greater number and are infiltrating into the tumor mass as compared to the other groups where they remain primarily on the periphery. The findings here elucidate a system where the IFN with 5Aza and vaccine components act in tandem to create a microenvironment more conducive to immune activity.

## Materials and methods

### Tumor model

6–12-week-old female C57BL/6 (Charles River, Wilmington, MA) mice were challenged with a lethal dose of B16F10 melanoma (5×10^4^ cells, >95% viability) administered intradermally on the mouse flank on day 0 of therapy (10, 11). Tumor size was recorded by calipers every 1–3 days as square mm (L × W). Mice were monitored for signs of distress in accordance with IACUC protocols.

### Vaccinations and therapeutics

**Figure 1:**
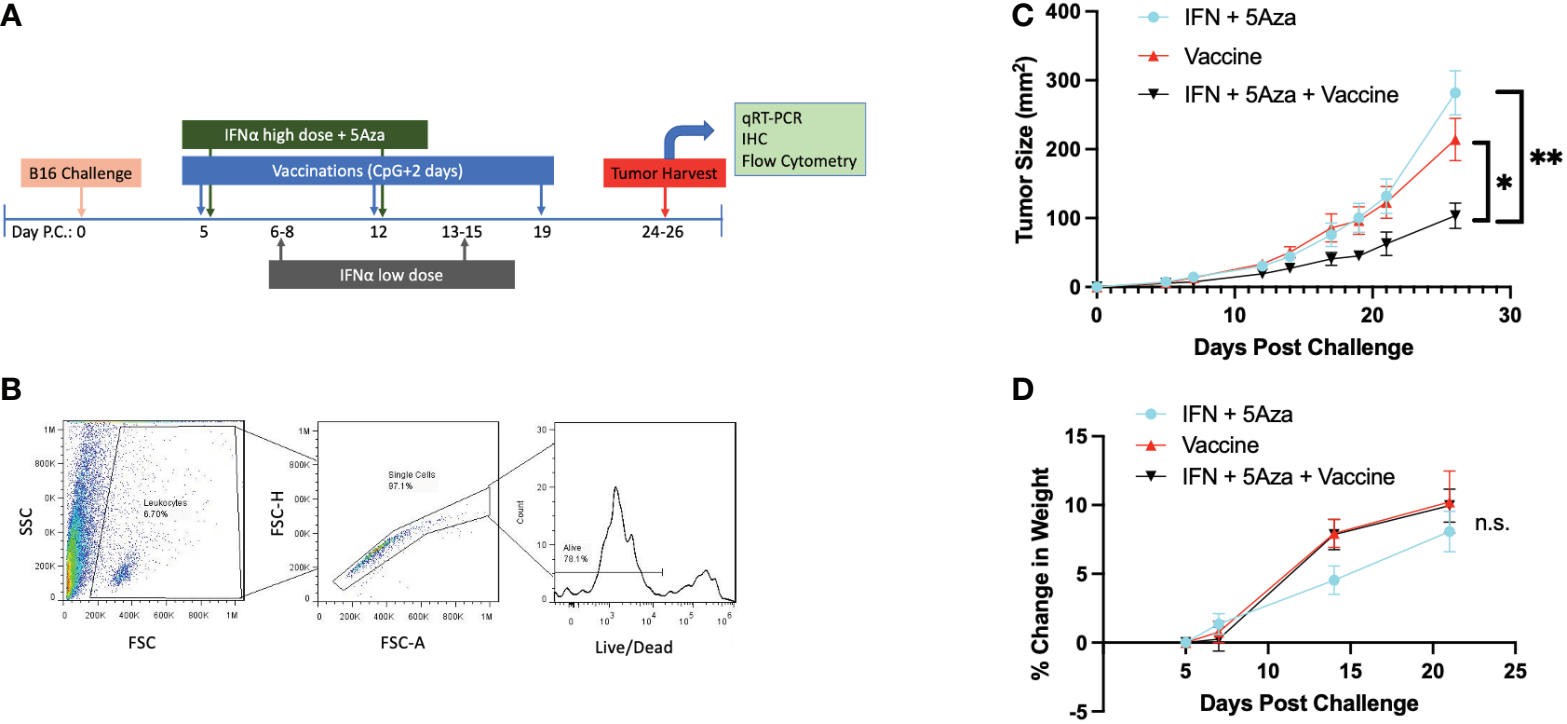
Experimental Design and General Characteristics. **(A)** Challenge and treatment outline. **(B)** Flow cytometry initial gating strategy. Potential leukocytes are gated by FSC vs SSC, followed by doublet discrimination, and live cell selection. All flow cytometry data in these studies utilize these three gates. **(C)** Tumor size over time across treatments. Data are represented as average of experimental means across all experiments that have data referenced in this study (N=5). Day 26 time point has significant differences by 2-Way Anova with Tukey’s test. *p<0.05; **p<0.01. **(D)** Change in mouse weight across groups and time. Analysis stopped at day 21 before tumor masses became a significant proportion of mouse weight. Data are representative of 6-14 mice across 2-3 independent experiments. Data analyzed by Mixed Effects Analysis with Tukey’s test. Relationships are not significant (n.s.).

Vaccine antigen is the MIP-3α-Gp100-Trp2 (tyrosinase-related protein 2) DNA construct in the pCMVe mammalian expression plasmid published here (11). Vaccination-grade plasmids were extracted from *E. coli* DH5-α (Invitrogen™ ThermoFisher Scientific, Waltham, MA) using Qiagen^®^ (Germantown, MD) EndoFree^®^ Plasmid Kits and were diluted with endotoxin-free 1xPBS. Vaccine DNA preps were verified by insert sequencing (JHMI Synthesis and Sequencing Facility, Baltimore, MD), spectrophotometry, and gel electrophoresis, and then administered at 50 μg/dose into the gasctocnemius muscle followed by *in vivo* electroporation, pulsing the muscle with the ECM 830 Electro Square Porator with 2-Needle Array Electrode (BTX Harvard Apparatus; Holliston, MA) under the following parameters: 106 V; 20 ms pulse length; 200 ms pulse interval; 8 total pulses (10). 50 μg ODN2395 Type C CpG (Innaxon LPS Biosciences, Tewkesbury, UK) was administered intramuscularly 2 days post-vaccination into vaccinated muscle. Recombinant mouse interferon alpha-A (IFNα, R&D Systems, Inc. Minneapolis, MN) was administered intratumorally as a series of doses: one high dose (10,000 units) followed by 2-3 days of low doses (1000-2000 units). InSolution™ 5 Aza 2′-deoxycytidine (5Aza, CalBiochem^®^, MilliporeSigma, Burlington, MA) was administered intraperitoneally at 1 mg/kg in 50 μl, at approximately 20 μg/mouse. **Figure 1A** outlines the therapy schedule.

### Lymphocyte extraction and flow cytometry

Tumor cell suspensions were prepared as previously described, with or without the Lympholyte M (Cedarlane Labs, Burlington, NC) purification step (10, 11). Briefly, tissue was extracted, kept cold, ground through a filter, washed, either purified or processed to lyse red blood cells, and used for downstream applications. In one experiment, the tumor cells were cryopreserved in 90% FBS 10%DMSO freezing media using isopropanol baths at -80°C prior to moving the samples to -150°C. Cells were quick-thawed and allowed to rest for 4 hours at 37°C before proceeding. Cells undergoing a freeze-thaw were only utilized for T-cell subtype analyses. If fewer than 10 live CD8+ T cells were measured, the samples were not included due to poor cell viability. Results of T-cell subtypes from cryopreserved cells were not significantly different from the remainder of the datasets. Tumor cell suspensions were stained in a 96-well V-bottom plate (Sarstedt, Inc., Newton, NC), with combinations of the following anti-mouse mAbs: PercPCy5.5 conjugated anti-CD3, CD11b-APC, Ly6G-FITC, Ly6C-Percp-Cy5.5 (eBioscience, Inc., San Diego, CA), FITC-CD8, NK1.1-PE, Live/Dead Near-IR (Invitrogen by Thermo Fisher Scientific, Carlsbad, CA), CD8-AF700, CD62L-APC, CD44-AF700, NK1.1 AF700, CD11c-PE, and CCR7 PE-Cy7 (Biolegend, San Diego, CA). The Attune™ NxT (Thermo Fisher Scientific, Waltham, MA) flow cytometer was utilized. Flow data were analyzed by FlowJo Software (FlowJo, LLC Ashland, OR) or Attune NxT Software v3.2.1 (Thermo Fisher Scientific, Waltham, MA). Tumors smaller than 25mm^2^ were not analyzed due to an insufficient amount of tissue. **Figure 1B** shows common initial gates: cells were gated on potential immune cells by FSCxSSC, screened out doublets and clumps, and selected for alive cells. %Alive refers to the percentage of all cells passing through these first three gates. Gates were formulated using full-minus-one (FMO) staining controls as reference.

### RNA extraction and qRT-PCR

Cross-sections of tumor weighing less than 100 mg were harvested. Tumor was minced as finely as possible, added to 1 ml Trizol^®^ (Ambion^®^ by Life Technologies, Carlsbad, Ca), and then homogenized by the Fisher Scientific™ PowerGen125 (Thermo Fisher Scientific, Waltham, MA). RNA was extracted according to the manufacturer’s protocol. The cDNA reverse transcription reaction utilized the SuperScript™ III First-Strand Synthesis System (Invitrogen ™, Waltham, MA), as per the manufacturer’s protocol. Real-time quantitative reverse transcription-PCR (qRT-PCR) was performed utilizing TaqMan^®^ Gene Expression Master Mix or Fast Advanced Master Mix and TaqMan^®^Gene Expression Assays (Applied Biosystems™ by Thermo Fisher, Halethorpe, MD) with probes specific for mouse GAPDH, XCL1, and CXCL10, utilizing the manufacturer’s protocols. Ct threshold was standardized across experiments, and the Ct statistic equated to the average of triplicate technical replicates. For analysis, ΔCt is calculated by subtracting the Ct value of the housekeeping gene GAPDH from that of the gene of interest. qRT-PCR was performed utilizing the StepOnePlus™ machine and software (Applied Biosystems™ by Thermo Fisher, Halethorpe, MD).

### Histology

Tumor cross-sections or whole tumors were fixed in 10% neutral buffered formalin. The samples were embedded in paraffin, cut in levels and adhered to slides, and then cuts from the same level were stained with hematoxylin and eosin (H&E) or labelled CD8 by immunohistochemistry (IHC) in parallel by the Sydney Kimmel Comprehensive Cancer Center Histology Core Facility (Baltimore, MD). All H&E and CD8 IHC cases were reviewed by a board-certified dermatopathologist (JCS) who was blinded during histologic scoring and evaluated for overall histologic appearance and degree of immune response. Immune infiltration was scored semi-quantitatively, with 0 for no inflammation, 1 for mild, 2 for moderate, and 3 for strong peripheral and intratumoral T-cell infiltration. Images were digitally brightened by 10%. Images are presented from the 10x objective, and zoomed images were digitally zoomed an additional 2x. Quantitative analysis of the infiltrating CD8+ cells was performed by two individuals blinded to the groups, each counting stained CD8+ cells across 10 random fields per sample (40x objective). The mean value of the 20 fields counted was utilized as the data point for analysis.

### Statistics and data

Tumor size, qRT-PCR, and flow cytometric analyses were statistically tested by one-way ANOVA with Tukey’s multiple comparison test if dataset distributions were approximately normal or with Dunn’s multiple comparison test if not. Normality was assessed by D’Agostino & Pearson test primarily or by Shapiro-Wilk test if the sample size was too small. Scatter plots were analyzed by simple linear regression with Spearman correlation coefficient test. Grouped experiments were analyzed by 2-way ANOVA with Sidak multiple comparison test. Microsoft^®^ Excel (Microsoft Corp, Redmond, WA) was used for database management. Prism 9 (GraphPad Software, Inc. San Diego, CA) was utilized for statistical analyses and figure creation. All error bars represent the estimation of the standard error of the mean, and all midlines represent the group mean. The significance level of α ≤ 0.05 was set for all experiments. Data provided in (**Supplementary file 1**).

## Results

### Tumor model

**Figure 1A** outlines the therapy schedule utilized both in this study and our prior work (10, 11). Importantly, the tumor growth phenotype remains consistent across experiments (**Figure 1C**), allowing the studies here to expand on previous work. Since our prior studies found that mice receiving no treatment did not survive to days 24-26 (10, 11), in the current study we decided to compare the three treatment groups to each other. At select time points, mouse weight was measured to ensure the therapeutic regimen did not induce excessive stress. A representative gating strategy indicating the initial steps of gating for samples is shown here as well (**Figure 1B**). All groups consistently gained weight over time, and there was no significant difference in weight change across the groups (**Figure 1D**).

### Tumor lysate chemokine expression

**Figure 2:**
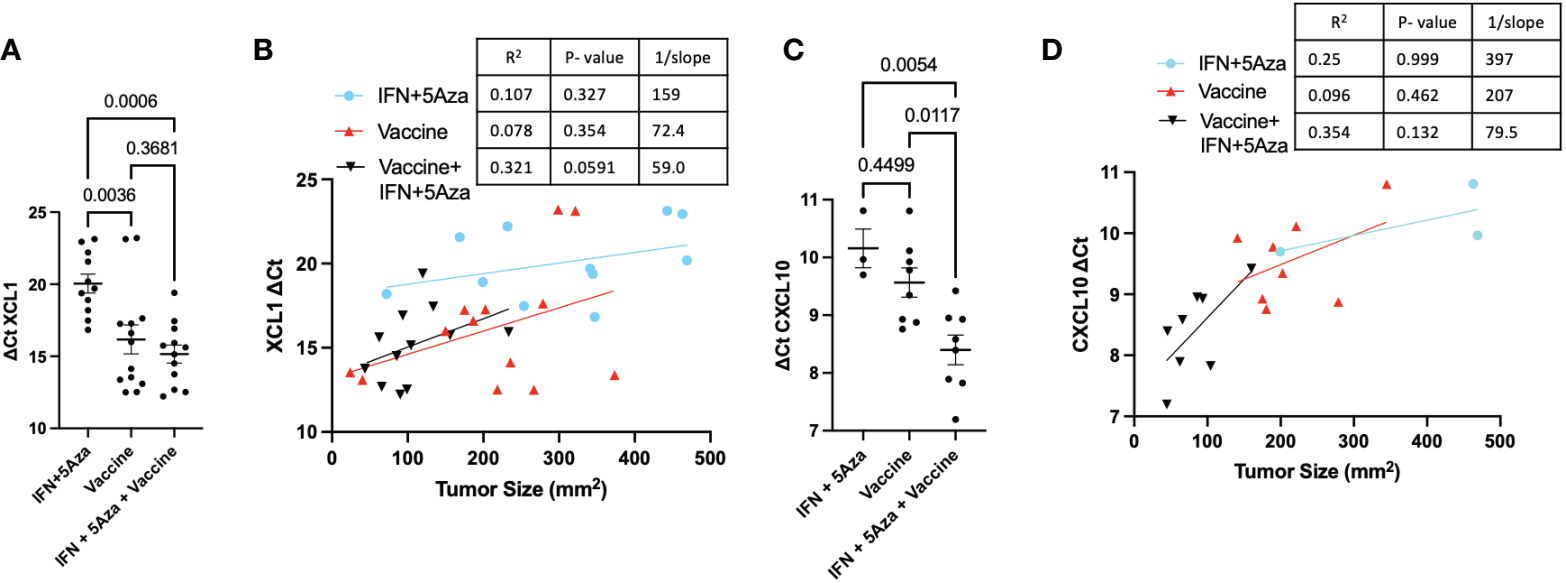
Tumor Lysate Chemokine RNA Expression. At harvest, RNA was Extracted from all or a cross-section of tumor and analyzed by qRT-PCR. Values are expressed as ΔCT normalized to GAPDH expression. **(A)** Expression of XCL1 across groups and **(B)** correlated to tumor size. **(C)** Expression of CXCL10 across groups and **(D)** correlated to tumor size. All data represent 2-3 independent experiments with sample sizes ranging from 3-5 per group per experiment. Group comparisons were tested by One-Way Anova with Tukey’s multiple comparisons test. Correlations analyzed by Spearman correlation coefficient test.

Our previously published data showed significant upregulation of CCL19 in mice receiving combination therapy (11). Since CCL19 has been implicated in homing CCR7+ immune cells to the lymph nodes (12), additional chemokines associated with immune cell infiltration were chosen for qRT-PCR analysis. XCL1, essential in attracting cross-presenting DCs (13), showed higher expression in the vaccine alone (p = 0.0036) and combination (p = 0.0006) groups compared to IFN + 5Aza (**Figure 2A**). XCL1 expression was also correlated with trends of reduced tumor size in the combination group (p = 0.0591, R^2^ = 0.321) (**Figure 2B**). CXCL10 is a primary recruiter of T cells (14), and its transcription levels were also significantly elevated in the combination group compared to both vaccine alone (p = 0.0117) and to IFN + 5Aza (p = 0.0054) (**Figure 2C**). Additionally, levels were correlated with trends of reduced tumor size in the combination group (p = 0.132; R^2^ = 0.354) (**Figure 2D**). In both cases, the combination group is the only group with an R^2^ above 0.3. Across all groups, XCL1 (R^2^: 0.53, p<0.001) and CXCL10 (R^2^: 0.584; p<0.001) also showed significant overall correlations.

### Natural killer and dendritic cells

**Figure 3:**
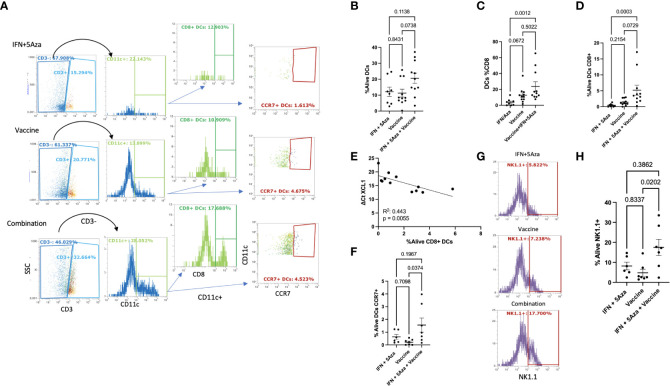
Dendritic Cell and Natural Killer Cell Tumor Infiltration. **(A)** Gating of DCs. Single, live, leukocytes (**Figure 1B**) were selected for CD3 negativity and CD11c positivity to determine the DC population. DC’s were analyzed for CD8 and CCR7 expression. Plots shown are representative samples from their groups. **(B)** %Alive analysis of total DCs. **(C)** Analysis of the percentage of DC’s that are CD8+ per group. **(D)** %Alive analysis of CD8+ DCs. **(E)** Correlation of XCL1 expression versus %Alive analysis of DC’s expressing CD8. **(F)** %Alive DC’s expressing CCR7. **(G)** Representative gating of NK1.1 positivity. **(H)** %Alive of NK1.1 positive cells. All data represent 2-3 independent experiments with sample sizes ranging from 3-5 per group per experiment, except panel E, which represents one experiment. Group comparisons were tested by One-Way Anova with multiple comparisons test (Tukey’s if approximately Gaussian **(F)** or Dunn’s if not **(B–D, H)**. Correlations analyzed by Spearman **(E)** correlation coefficient test.

Emerging evidence suggests that DC- and NK- cell infiltration into the TME aids in mounting an effective anti-tumor response (15, 16). To understand the cellular makeup of the TME, we performed flow cytometric analysis on tumor lysates. **Figure 3A** shows the gating strategy of representative samples. While there was too much variation to achieve statistical significance, a trend of increased intratumoral infiltration of CD3-CD11c+ DCs was observed between the combination group and the IFN + 5Aza (p = 0.1138) and vaccine alone (p = 0.0738) groups (**Figure 3B**). Interestingly, the percentage of CD8+ DCs, representing inflammatory and cross-presenting DCs (17), was modestly increased following vaccination alone (p = 0.0672), and significantly increased following combination treatment (p = 0.0012) relative to IFN+5Aza (**Figure 3C**). However, only the combination group had enhanced levels infiltrating the tumor compared to the IFN + 5Aza group (p = 0.0003; **Figure 3D**). Correlation between XCL1 expression and the presence of CD8+ DCs was also highly significant (p = 0.0055, R2 = 0.443) (**Figure 3E**). Additionally, the upregulation of CCL19 and CCR7 (11) seen in the combination group, and the fact that CCL19 binds CCR7 directed us to investigate the percent of CCR7+ DCs present in the tumor lysate (**Figure 3F**), which were significantly higher in the combination group (p = 0.037) when compared to the vaccine group. NK cell numbers were also analyzed (representative gating in **Figure 3G**) and were significantly higher in the combination therapy group when compared to vaccine alone (**Figure 3H**, p = 0.0202).

### Myeloid-derived suppressor cells

**Figure 4:**
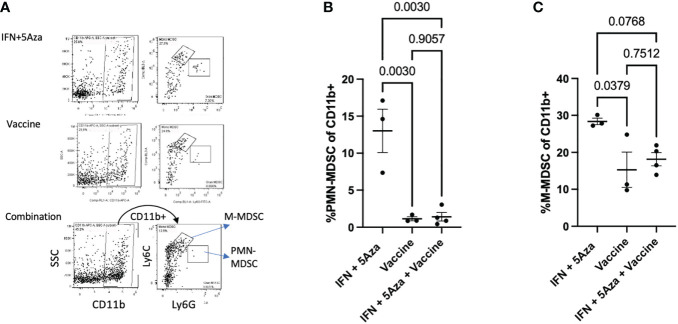
MDSC analysis. **(A)** Gating structure. Cells were selected as CD11b+ and then divided into PMN-MDSCs (Ly6G hi/Ly6C lo) and G-MDSCs (Ly6C hi, Ly6G lo). Plots shown are representative samples from their groups. **(B, C)** Grouped analysis as a percentage of CD11b+ cells of **(B)** PMN-MDSCs and **(C)** M-MDSCs. Data are representative of one experiment with 3-4 mice per group and are tested by by One-Way Anova with Tukey’s multiple comparisons test.

A category of innate immune cells known as MDSCs (myeloid derived suppressor cells) is often present in the TME and disables an effective anti-tumoral response by potentiating immunosuppressive activity (17). To analyze whether these cells were present in the TME, we performed flow cytometry on the tumor lysate; both classes of murine MDSCs were analyzed and included M-MDSC (monocytic MDSCs) and PMN-MDSC (polymorphonuclear or granulocytic MDSCs). **Figure 4A** shows the representative gating strategy. The percent of both PMN-MDSCs (**Figure 4B**) and M-MDSCs (**Figure 4C**) were reduced in the vaccine alone and combination therapy groups. The differences between mice receiving the vaccine alone compared to IFN + 5Aza were significant for PMN-MDSCs (p = 0.003) and M-MDSCs (p = 0.0379). When comparing combination therapy to IFN + 5Aza, there was significant reduction of PMN-MDSCs (p = 0.003) and a trend towards reduction of M-MDSCs (p = 0.0768).

### Tumor infiltrating lymphocytes

**Figure 5:**
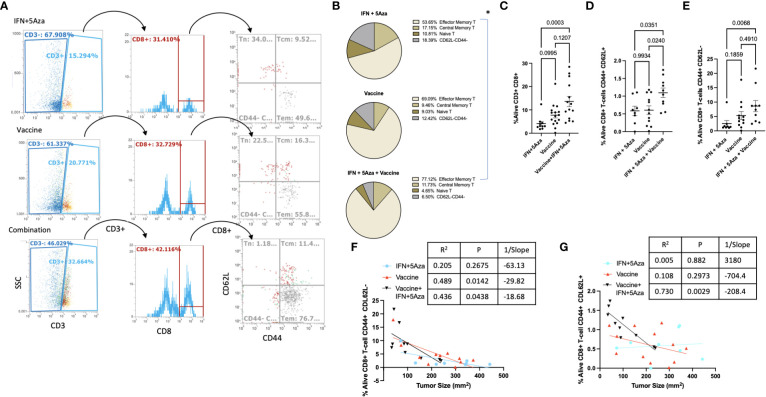
T-cell subtype analysis. **(A)** Gating structure. CD3+CD8+ cells are divided by CD44 and CD62L expression patterns. CD44+CD62L- are T effector/effector memory cells, CD44+CD62L+ are central memory T-cells, and CD44-CD62L+ are naïve T-cells. Plots shown are representative samples from their groups. **(B)** For each group, proportions of the three primary subtypes are plotted by pie chart. **(C–E)** %Alive analysis of tumor infiltrating populations of **(C)** all CD8+ T-cells, **(D)** Central Memory CD8+ T-cells, and **(E)** effector/effector memory CD8+ T-cells. **(F, G)** Correlation analysis between **(F)** CD8+ Tem or **(G)** CD8+ Tcm and tumor size. Data are representative of 3-4 independent experiments with 10-16 total mice per group. Groups were tested by One-Way Anova with multiple comparisons: Tukey’s test **(B–D)** or Dunn’s test **(E)**. Correlations tested by Spearman correlation coefficient test.

Our previous data indicated increased levels of CD8+ T cells in the combination therapy group (10, 11), and findings from this study confirmed those results. **Figure 5A** shows the representative gating strategy used to stratify CD8+ T cells, and total CD8+ T cells were significantly enriched in the combination group compared to IFN + 5Aza (**Figure 5C**, p = 0.0003) and trending towards significance in vaccine alone (p=0.1207). To understand differences in CD8+ T-cell composition, CD3+ CD8+ T cells were further gated on CD44 and CD62L to categorize effector (CD44+ CD62L-), naive (CD44- CD62L+), and central memory (CD44+ CD62L+) T cells (**Figure 5A**). When total CD8+ T cells were stratified based on percentage, naïve and double negative (CD44- CD62L-) T cells were qualitatively lower in the combination therapy group compared to mice receiving either IFN + 5Aza or vaccine alone, and the combination group was primarily composed of central memory and effector memory T cells (**Figure 5B**). Importantly, the combination therapy group showed significantly higher amounts of effector memory CD8+ T cells (**Figure 5E**, p = 0.0068) compared to mice receiving IFN + 5Aza, and also showed increased numbers of central memory T cells relative to both mice receiving vaccine alone (p = 0.024) and IFN+ 5Aza (p = 0.0351) (**Figure 5D**). Furthermore, the correlations between decreased tumor size and increased CD8+ T effector memory cell or CD8+ T central memory cell infiltration into the TME in the combination treatment group (p = 0.0438, R^2^ = 0.436; **Figure 5F**, p = 0.0029, R^2^ = 0.73; **Figure 5G** respectively) were significant.

### Immunohistochemistry

**Figure 6:**
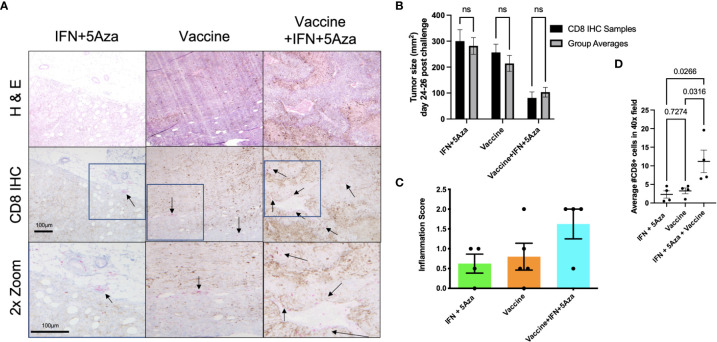
CD8 Immunohistochemistry (IHC). **(A)** IHC of tumor sections. CD8+ cells were stained with pink color to differentiate from brown melanin background. Representative images were selected and scored by group-blinded dermatopathologist from slides stained from 4 samples per group across two independent experiments. Top two rows utilized 10x objective. Bottom row images were digitally zoomed 2x from boxes in middle row. Arrows are pointing to select clusters of CD8+ cells. **(B)** Tumor size comparison of CD8 IHC samples to group averages across experiments, tested by 2-way Anova with Sidak multiple comparison test; n.s. = not significant. **(C)** Semi-quantitative tumor inflammation scores. **(D)** Average of CD8+ cell counts from twenty random and blinded fields per sample from parenchymal regions using a 40x objective, tested by One-way Anova with Tukey’s multiple comparison test.

A representative sample of four tumors per group across two experiments were selected for microscopy analysis. Tumor cross-sections were mounted onto slides and stained for H&E and CD8 by immunohistochemical methods. **Figure 6A** shows representative images with selected clusters of CD8+ cells pointed to with arrows, with areas of interest outlined in boxes and zoomed an additional 2-fold to more clearly show the stained cells. In all the IFN + 5Aza samples and three out of four vaccine-only samples, CD8+ cells were visualized primarily around peritumoral vessels but not infiltrating the tumor mass (arrows, **Figure 6A** left and middle). Three out of four samples from the combination group showed substantial infiltration of CD8+ cells into the tumor mass (arrows, **Figure 6A** right). **Figure 6B** provides evidence that our sampling was representative, as the tumor size averages did not significantly differ from across group means.

Additionally, the samples were scored in a group-blinded fashion for level of inflammation ranging from 0-3 (**Figure 6C**). The IFN + 5Aza group showed little inflammation with one out of four samples scored as 0, and three with minimal inflammation, scored as 0+ to 1. The vaccine alone group showed higher variability, with one tumor lacking any inflammation (scored 0), two showing only peripheral CD8+ cell accumulation (scored 0+ to 1), and one with peripheral accumulation and intratumoral penetration (scored 2). The combination group resulted in the highest proportion of cases with both peripheral CD8+ cell accumulation and CD8+ cell penetration, with three out of four samples scored at 2.

To provide quantitative analysis of the infiltration of CD8+ cells into the tumor mass, twenty total randomly selected parenchymal 40X objective microscope fields per sample were counted by two group-blinded individuals. **Figure 6D** shows that the combination group had significantly more CD8+ cells that infiltrated the tumor parenchyma as compared to IFN+5Aza (p=0.0266) and to vaccine alone (p=0.0316).

## Discussion

Treatment of late-stage tumors, including metastatic melanoma, has historically been challenging as elements within the TME interfere with immune cell infiltration and limit an effective anti-tumoral response (18). Combining traditional therapies with other treatment options including immunotherapy, however, has yielded impressive results and improved prognosis (19–21).

In our study, we utilized a MIP3α fused vaccine targeting two common melanoma antigens, gp100 and trp2, in combination with 5Aza and IFN. Previously published data indicated high efficacy of this combination therapy compared to vaccine or 5Aza and IFN alone, as manifested by greater median survival time and reduced tumor burden in mice given the combination therapy. Our work also indicated that this group had greater CD8+ T-cell infiltration into the TME, as well as significant CCL19 upregulation (11). These findings prompted this study, which reinforced the CD8+ T-cell data (**Figure 5C**), but also introduced the possibility of DC and NK cell anti-tumoral action in the TME (**Figure 3**) and highlighted the immunosuppressive role MDSCs may play in dampening immune responses (**Figure 4**). Additionally, we also noted upregulation of the chemokines CXCL10 and XCL1 (**Figure 2**); XCL1 in the combination group was upregulated relative to the IFN+5Aza group, and CXCL10 in the combination group was upregulated relative to both vaccine alone and IFN + 5Aza groups. Furthermore, stratification of CD8+ T-cells revealed increased percentages of effector memory and central memory T-cells in the combination group (**Figures 5E, D**), which were highly correlated with decreased tumor burden (**Figures 5F, G**). Importantly, results from this study further define immunological mechanisms underlying the synergism seen previously of all combination therapy components in enhancing survival and anti-tumoral activity (11).

Optimally activated CD8+ T cells are critical to tumor control, and the presence of both effector memory and central memory CD8+ T cells has also been correlated with an effective anti-tumoral response. Our findings indicate that the combination therapy can elicit an effective memory response, which is critical to remission (22, 23). In many solid tumors including melanoma, immune infiltration into the TME is depressed and leads to a limited immune response ineffective in killing the cancer cells (18). A high expression of chemokines related to DC, NK, and T-cell recruitment within the TME has been associated with greater influx of these cells into the tumor and consequently, better prognosis (15, 16, 24). We saw heightened expression of CCL19 (11), CXCL10, and XCL1 in mice treated with the combination therapy, suggesting the creation of an environment favoring greater immune cell influx. CCL19 binds to CCR7 on a multitude of cell types including DCs and T-cells, whereas CXCL10 is a is a canonical chemokine for attracting T-cells (14), and its upregulation is correlated with smaller tumor size. XCL1, on the other hand, is secreted by activated NK cells and CD8+ T-cells and is part of the Th1 response. It binds XCR1, present on conventional DCs type 1 (cDC1), NK cells, and CD8+ T-cells (13). Bottcher et al. (25) found that XCL-1 and CCL5 secreting NK cells promoted cDC1 infiltration into the TME, which was correlated with higher survival and better prognosis, and other studies have also noted the anti-tumoral role facilitated by DC-NK crosstalk (26). It is possible that these processes are also occurring in our system.

These results, when taken together, indicate that the combination therapy is integral in creating an effective anti-tumoral environment composed overall of CD8+ T-cells of memory phenotypes, CD8+ DCs, and NK cells. We believe that this occurs primarily by increased immune cell trafficking into the TME, as seen by the IHC (**Figure 6**). This increased immune infiltration into the TME requires the presence of all three components of the combination therapy and is likely not attributed to a singular element. This is best demonstrated by the IHC data, where the combination group has consistently higher levels of inflammation and of CD8+ cells infiltrating the tumor parenchyma compared to the IFN+5Aza and vaccine alone groups. The recruitment of these cells is likely due to the upregulation of CCL19, CXCL10 and XCL1, among other potential untested targets, which enable cells to respond to the chemokine gradient and infiltrate the TME.

Overall, our results suggest that the establishment of an effective tumor-killing environment composed of favorable cell types, such as DCs, CD8+ memory T-cells, and NK cells, as well as important chemokines, including CCL19, CXCL10, and XCL1 relies on all aspects of the combination therapy. This study provides evidence for well-designed cancer vaccines as an important arm of combined therapy and supports the use of combination therapies in the clinic for metastatic tumors. Future studies will aim to further define the TME, and the protective immune responses elicited by combination treatment. A lung metastatic model of the disease will also be incorporated to extend our knowledge of treatment efficacy.

## Data availability statement

The original contributions presented in the study are included in the article/supplementary material. Further inquiries can be directed to the corresponding author.

## Ethics statement

The animal study was reviewed and approved by the IACUC of the Johns Hopkins University under Protocols #MO16H147 and MO19H139.

## Author contributions

JG and AS performed and analyzed the experimental studies and co-wrote the manuscript. KF performed confirmatory studies and XCL1 qRT-PCR. KL, AK, SA, YH, and SK assisted with animal studies, tissue processing, and assays. CS, IC, and TW assisted with manuscript revision and microscope analyses. JS performed the immunohistochemical analysis. PK and RM contributed to the conception and design of the experiments and the writing of the manuscript. All authors contributed to the article and approved the submitted version.
